# Semiautomatic Segmentation of Glioma on Mobile Devices

**DOI:** 10.1155/2017/8054939

**Published:** 2017-06-27

**Authors:** Ya-Ping Wu, Yu-Song Lin, Wei-Guo Wu, Cong Yang, Jian-Qin Gu, Yan Bai, Mei-Yun Wang

**Affiliations:** ^1^School of Electronic and Information Engineering, Xi'an Jiaotong University, Xi'an, Shaanxi 710049, China; ^2^Collaborative Innovation Center for Internet Healthcare and School of Software and Applied Technology, Zhengzhou University, Zhengzhou, Henan 450001, China; ^3^Henan Provincial Clinical Big Data Analysis and Service Engineering Research Center, Zhengzhou University People's Hospital and Henan Provincial People's Hospital, Zhengzhou, Henan 450001, China; ^4^Department of Radiology, Zhengzhou University People's Hospital and Henan Provincial People's Hospital, Zhengzhou, Henan 450001, China

## Abstract

Brain tumor segmentation is the first and the most critical step in clinical applications of radiomics. However, segmenting brain images by radiologists is labor intense and prone to inter- and intraobserver variability. Stable and reproducible brain image segmentation algorithms are thus important for successful tumor detection in radiomics. In this paper, we propose a supervised brain image segmentation method, especially for magnetic resonance (MR) brain images with glioma. This paper uses hard edge multiplicative intrinsic component optimization to preprocess glioma medical image on the server side, and then, the doctors could supervise the segmentation process on mobile devices in their convenient time. Since the preprocessed images have the same brightness for the same tissue voxels, they have small data size (typically 1/10 of the original image size) and simple structure of 4 types of intensity value. This observation thus allows follow-up steps to be processed on mobile devices with low bandwidth and limited computing performance. Experiments conducted on 1935 brain slices from 129 patients show that more than 30% of the sample can reach 90% similarity; over 60% of the samples can reach 85% similarity, and more than 80% of the sample could reach 75% similarity. The comparisons with other segmentation methods also demonstrate both efficiency and stability of the proposed approach.

## 1. Introduction

Glioma is a prevalent fatal brain disease, accounting for about 50% of instances among all intracranial tumors, which is also the major malignancy brain disease with the highest mortality and morbidity. Traditionally, the determination and staging of glioma are mainly based on a radiologist's experience and intuition, leading to poor diagnosis stability and reliability. Through accurately and reliably converting medical images into quantified digital features, radiomics provides an effective solution for automatic detection and determination of glioma by describing the microenvironment of tumor lesion [1–3]. In addition, it has been shown to be effective in computer-aided diagnosis and computer-assisted surgery and radiotherapy as well as medical research of glioma patients by extracting personalized features for individual patient.

Radiomics typically uses machine learning to train a model for classification or prediction. The segmentation of the region of interest (ROI) is the most critical step, which is also the foundation of all subsequent analyses. In practice, existing glioma segmentation is hard to be applied in clinical routines because of the heterogeneous nature of glioma and the image acquisition procedures. Firstly, due to the characteristics of glioma, the tumor tissue shows no clear boundaries with normal tissues [4]. The gray levels between different tissues have similar gray values in the MRI images. In addition, glioma's inherent complexity exhibits complex pathological changes (including hemorrhage, necrosis, and edema appearance). In particular, the nature of the tumor's subregion is the gene mutation, so the heterogeneous nature is chaotic [5]. Secondly, the glioma image contains Gaussian white noise and bias field caused by coil magnetic of the equipment, which can be expressed as low-frequency global multiplicative noise, and therefore, these images show no uniformity in the same tissue. Partial volume effect resulted from equipment resolution indicating that the gray level of pixel reflects the average gray level of voxel. Active or inactive movement of patients between acquisition processes has negative impact on images. For the above reasons, glioma images show blurry edges and uneven gray levels, thus making accurate, repeatable, and stable segmentation a challenging task. Indeed, accuracy and robustness of existing glioma segmentation algorithms are insufficient; thus, radiologists are still needed for assisted image segmentation to ensure the quality of radiomics.

On the other hand, with the popularity of mobile devices as well as the development of wireless technology, smartphones and tablets have become routine office tools. An increasing number of doctors started to use mobile devices to handle clinical tasks, such as viewing medical images and submitting diagnostic advice. Thus, in the context of semiautomatic segmentation, it is desirable to allow doctors to do supervised segmentation in their convenient time. Due to the limited computing power of mobile devices, it is challenging to directly process the raw image data on the devices [6–8]. In order to improve the experience of interactive segmentation on low bandwidth and often unstable wireless network, we often use a client/server model where medical images are preprocessed on the server while doctors supervise segmentation on the mobile client. This framework thus requires small data transfer between the mobile client and server and a fast segmentation algorithm on the mobile client.

The contributions of this paper include the following:

1. We adapted the multiplicative intrinsic component optimization (MICO) algorithm [9] to denoise and presegment tissues for further processing. The original MICO often produces blurry edges, making the preprocessed images less usable for radiologists. Thus, we replaced its fuzzy membership function with a binary function to obtain clear edges. We also replaced the fixed iteration number with an empirically derived threshold for algorithm termination, which reduces preprocessed image size for wireless transfer from the server to mobile client. On the mobile device, we used the multiseed region-growing (MSRG) segmentation algorithm to automatically calculate the region of interests based on seeds chosen by the doctors.
2. We experimentally evaluated the proposed approach and compared with popular segmentation algorithms to show its effectiveness and consistency. In addition, we implemented and evaluated the mobile application to show that the MSRG algorithm runs efficiently on mobile devices that allows for good interaction performance.

## 2. Related Works

According to the degree of required human interaction, brain tumor segmentation methods can be classified into three categories [10], manual segmentation, automatic segmentation, and semiautomatic segmentation. Manual segmentation is to label tumor lesions manually slice-by-slice by a radiologist which is time consuming and tedious. Besides, it is challenging to repeat due to its strong subjective and heavy workload, which is also of limited use for objective quantitative analysis. Automatic segmentation is controlled entirely by the algorithm, and there is no requirement of interaction with the high segmenting speed. However, due to the severe inherent heterogeneity of tumors together with bias field and noise, the accuracy of the automatic segmentation algorithm is often poor. In contrast, semiautomatic methods label the ROI with less interaction, adapt to different images and needs, and also achieve high accuracy and fast speed. Therefore, semiautomatic segmentation can balance the contradiction of segmentation accuracy and high labor intensity. Experiment results obtained by Parmar et al. [11] show that radiomics features extracted from semiautomatic segmentations had significantly higher reproducibility compared to that of extracted from the manual segmentations. To obtain stable radiomics features, the key is to reduce human interaction and simultaneously guarantee the accuracy and reproducibility as well as stability. Currently, the gold standard of glioma image segmentation does not exist. In clinical practice, two radiologists are often used to evaluate the segmentation results. Thus, reproducibility and stability are more significant in comparison with the accuracy.

The brain tumor segmentation algorithm can mainly be divided into the following categories [12]: region-based (or edge-based) method, classification and clustering method, and methods with constraints, as well as some hybrid methods.

Region growing is a classic region-based segmentation method, adding a pixel to the collection with similar properties to the seed point and iterative increase collection until achieving the target region segmentation. The key of region growing is to design the rules of measure similarity as well as the rules of growth. Rexilius et al. [13] initialized a region-growing algorithm with a tumor map, which was obtained from a multispectral histogram model adaptation. Bendtsen et al. [14] used the region-growing method presenting a semiautomatic segmentation algorithm LuTA for lung cancer and also apply it to analysis and evaluation of tumor volume. Gu et al. [15] expanded the single-click ensemble segmentation on the basis of LuTA and reduced the interactions in semiautomatic segmentation through multiple initial seeds produced in the core area, the external seeds produced by the 3-dimensional view expansion along 24 directions, and the stable tumor segmentation area generated by region-growing algorithm iteration. Due to the requirement of manually specifying the seed point and being sensitive to noise, the segmentation results obtained through the multiseed region-growing algorithm are likely to contain lots of holes, which generally require being combined into the segmentation results based on the circumstances.

The edge-based method uses the dramatic changes of gray value in the edge of the object. An active contour model (ACM) can use prior knowledge to solve image segmentation problems. Sachdeva et al. [16] made use of content-based intensity and texture patterns to evolve an active contour towards the tumor boundary in different MRI modalities. A level set segmentation algorithm expresses the evolving curve impliedly as the zero level of high dimension level set function, which possesses excellent theoretical foundation, can rapidly expand to 3-dimensional segmentation, and is extensively applied in glioma segmentation [17–19]. However, due to the heterogeneity of glioma and unclear boundary between tissues, level set segmentation has faced the problem of edge overflow problem, requiring further improvement for glioma.

Classification or clustering methods make use of voxel-wise intensity and texture features to segmentation, which is based on simple and intuitive idea that objects in the same class have small distance and objects between classes have broad distance. Ruan et al. [20] used SVM to segment brain tumors, which can only be handled with the lower number of modalities and one tumor region due to the binary classification characteristic of SVM. Deep neural networks (DNNs) were proposed to automatic brain tumor segmentation [21–23]. Havaei et al. [21] used a tailored convolutional neural network to segment glioblastomas. Zikic et al. [24] applied decision forest classification with context-aware features and an additional generative model as an input to identify tumor subcompartments from multimodal images. In addition to the algorithm simply using voxel-wised information, there have been numerous methods which attempt to use additional information to improve segmentation result [25–27]. Li et al. [9] proposed the multiplicative intrinsic component optimization (MICO) algorithm, which can be used to correct bias field and segment normal human tissue at the same time. To the best of our knowledge, there is no example applying the MICO algorithm into the pretreatment of glioma segmentation.

Although there exist many segmentation methods, tumor segmentation is still specifically designed for specific tasks and the general solution has not yet formed. Segmentation results have shown a great relationship with the initial state and parameters of an algorithm. As mentioned before, in radiomics applications, reproducibility and stability are more important compared to accuracy. MICO [9] is an efficient algorithm for normal human tissue segmentation; this paper uses a modified MICO algorithm to preprocess a glioma medical image on the server side, and then the doctors supervise the segmentation process on mobile devices. The preprocessed images have small data size and simple structure, which allows the follow-up steps to be processed on mobile devices with low bandwidth and limited computing performance. Through this way, a stable and reproducible method for the semiautomatic segmentation of glioma in the mobile environment is obtained.

The remainder of the paper is structured as follows: in Methods, the authors present the methods used in segmentation such as preprocessing on the server side, segmentation with multiseeds on mobile devices, and postprocessing like holes filling. In Results and Discussion, this paper describes the segmentation performance and analyzes the advances and unsuitable scenes of the method. Finally, in Conclusions, the authors conclude the work and give an outlook on future work.

## 3. Methods

**Figure 1:**
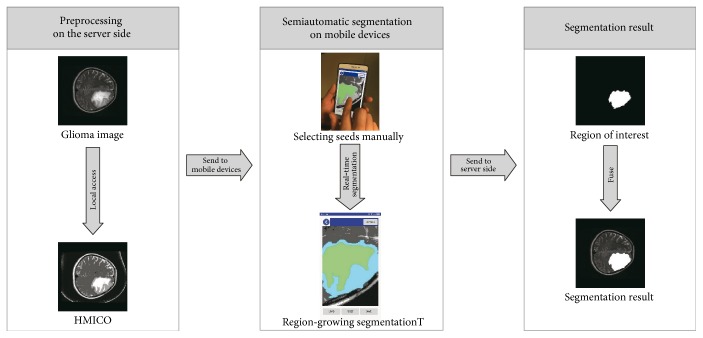
Segmentation algorithm flowchart.

In this work, the authors use the client/server framework to implement the segmentation of glioma. On the server side, a multiplicative intrinsic component optimization algorithm is adjusted to be suitable for glioma medical images and to enable that it can denoise and presegment tissues within which the same tissue voxels have the same brightness for further processing. And then, due to the special nature of preprocessed results, the radiologist can complete segmentation on the mobile devices just by manually specifying seed at any position within the ROI. The whole process of our method is shown in Figure 1.

### 3.1. Preprocessing on the Server Side

The preprocessing algorithm denoises and presegments tissues from the glioma imaging which is implemented by MATLAB® R2015b (Version: 8.6.0.267246) on a Dell Precision Tower 5810 workstation with Windows 10 Enterprise (x64) which CPU is Intel Xeon E5-1620V3@3.5GHz (8 CPUs) and has 16G memory.

Similar to other medical images, the noise of glioma images mainly derives from the following two aspects: one is the random noise, which is assumed as the Gaussian white noise in this paper, and the other is the bias field effect caused by heterogeneous magnetic field of the equipment coil, which manifests as low frequency multiplicative noise with global gradient. Therefore, the image model can be described using

(1) $$\begin{array}{c} I\left(x\right)=b\left(x\right)J\left(x\right)+n\left(x\right), \end{array}$$

where *I*(*x*) is the intensity of the observed image at voxel *x*, *b*(*x*) is the bias field, *J*(*x*) is the true image, and *n*(*x*) is the Gaussian white noise with zero mean.

It is discovered through observation that the glioma image consists of normal tissues such as protein, gray matter, and cerebrospinal fluid, as well as abnormal tissues like the accompanying edema, and all tissues are well defined. Without the loss of generality, it is assumed that the same tissues in the image has consistent brightness, and the boundaries between different types of tissues are clear and do not overlap. Based on this assumption, we assume that glioma images contain *N* piecewise constant regions. Noise energy is defined using (2) [9], and we can reach balance when the energy is minimum.

(2) $$\begin{array}{c} \begin{array}{c} F\left(u,c,w\right)=\int_{\Omega }\;|\;I\left(x\right)-w^{T}G\left(x\right)\overset{N}{\underset{i=1}{\sum }}c_{i}u_{i}\left(x\right)\;|\;{}^{2}dx=\int_{\Omega }\overset{N}{\underset{i=1}{\sum }}{\left|I\left(x\right)-w^{T}G\left(x\right)c_{i}\right|}^{2}u_{i}\left(x\right)dx, \end{array} \end{array}$$

where *w* = (*w*_1_,…,*w*_*m*_)^*T*^, *G*(*x*) = (*g*_1_(*x*),…,g_*m*_(*x*))^*T*^ is polynomial basis functions, *b*(*x*) = *w*^*T*^*G*(*x*), *c*_*i*_ is the gray level of the *i*-th tissues in the image, and *u*_*i*_ is an array indicating the *i*-th tissue or not, if the voxel is *i*-th tissue then corresponding cell sets are 1 and 0 otherwise and ∑*u*_*i*_ = 1, *J*(*x*) = ∑_*i*=1_^*N*^*c*_*i*_*u*_*i*_(*x*).

Unlike the original, MICO produces blurry edges, making the preprocessed images less usable for radiologists. The authors take off the fuzzy membership functions from origin MICO update functions in order to get clear boundary of glioma. After doing that, the result has a simpler structure and can reduce the size of data transferred from the server to the client and simplify the display and region-growing segmentation algorithm on a mobile which has a small screen as well as limited computing performance. To be compared with the origin MICO, the authors call this specific MICO as HMICO, which means hard edge MICO in the rest of the part of this paper. In this work, the authors use a gradient descent algorithm to optimize *u*, *c*, *w* by minimizing (2), fixing two parameters to optimize the third parameter.

(3) $$\begin{array}{c} \begin{array}{c} {\hat{c}}_{i}=\frac{\int_{\Omega }I\left(x\right)b\left(x\right)u_{i}\left(x\right)dx}{\int_{\Omega }b^{2}\left(x\right)u_{i}\left(x\right)dx},i=1,\ldots ,N,\\ \hat{w}={\left(\left(\int_{\Omega }G\left(x\right)G^{T}\left(x\right)\left(\overset{N}{\underset{i=1}{\sum }}c_{i}^{2}u_{i}\left(x\right)\right)dx\right)\right)}^{-1}\int_{\Omega }G\left(x\right)I\left(x\right)\left(\overset{N}{\underset{i=1}{\sum }}c_{i}u_{i}\left(x\right)\right)dx,\\ {\hat{u}}_{i}=\left\{\begin{array}{l} 1,i=i_{\min}\left(x\right)\\ 0,i\neq i_{\min}\left(x\right) \end{array}\right.,i_{\min}\left(x\right)=\arg\;\min\left\{\delta_{i}\left(I\left(x\right)\right)\right\}. \end{array} \end{array}$$

A large number of experiments prove that an energy-minimizing algorithm can be quickly converged in a finite number of steps. According to general experience, the algorithm converges to an available accuracy within 5–20 steps, and the origin MICO algorithm sets 20 times as default steps. To ensure the stability of the algorithm while reducing the number of iterations, after every iteration, (4) was used to calculate the energy descent rate Δ_*F*_, then terminate iteration when Δ_*F*_ is reaching a certain threshold value.

(4) $$\begin{array}{c} \Delta_{F}=\frac{\left(F_{n-1}-F_{n}\right)}{F_{n}}. \end{array}$$

### 3.2. Semiautomatic Segmentation on Mobile Devices

**Figure 2:**
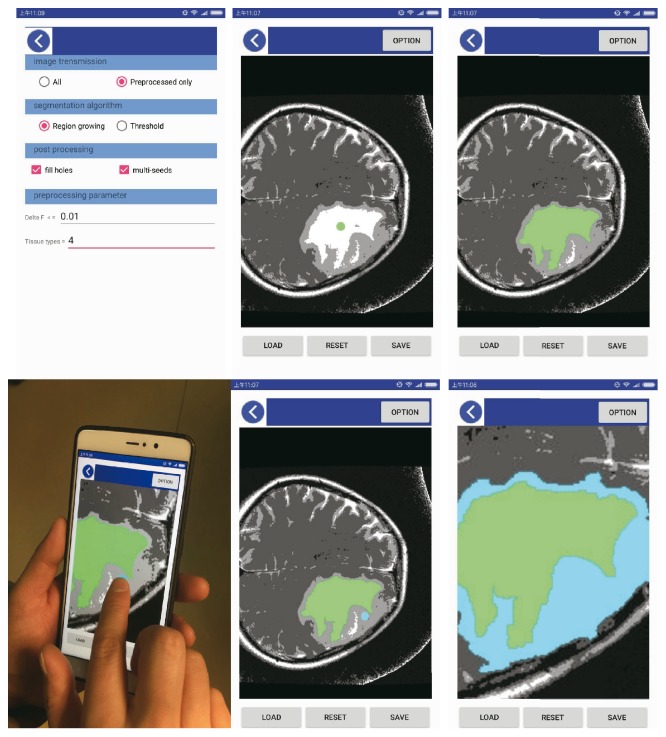
The user interface on the mobile device.

After preprocessing, a semiautomatic segmentation algorithm was implemented using region-growing segmentation with multiseeds on a mobile device. We use Java as the programming language and Android studio 2.2.3 as the GUI editor and test phones including MI 5S plus and HUAWEI P9 whose operating system is based on Android5.0. The user interface on the mobile device is shown in Figure 2.

Glioma images are pretreated by HMICO algorithms on the server side; the *N* kinds of tissues in the images have been labeled. The complicated growth pattern and the presence of heterogeneity in glioma images render regions with heterogeneous brightness in the tumor being divided into other regions by mistake. It can be found through observing the tumor region that the majority of tumor region has relatively uniform brightness, which has become uniform brightness after pretreatment; the radiologists only need to select the seed-growing points in the region, and they can obtain the tumor region of interest through a region-growing algorithm. In the case of the tumor region being pretreated as multiple adjacent regions due to heterogeneity, the uniform region of interest can be obtained through multiseed growing. The steps of region-growing segmentation with multiseeds are shown as follows:

 

(1) The radiologists set seed points and push these seeds into a stack.

(2) Pop up the seed-growing point (*x*0, *y*0) from the stack and take the 8 neighborhood pixels (*x*, *y*) into consideration with (*x*0, *y*0) being the center. If (*x*, *y*) satisfies the growth criteria, then combine (*x*, *y*) with (*x*0, *y*0) into the same region and push (*x*, *y*) into the stack.

(3) End the growth, if the stack is empty, and implement step 2.

Due to the caused heterogeneity, after the region-growing algorithm, segmentation result may contain many subareas in the main ROI area. These subareas can be well segmented through multiseeds, causing large workload for radiologists. Besides, it is worth noting that these subareas almost completely are contained in the main ROI area, so filling these holes (the area ratio is smaller than the threshold value) can obtain the complete area of the ROI.

## 4. Results and Discussion

In the experiment, the HMICO algorithm on the server side is implemented in Matlab using Δ_*F*_ ≤ 0.01 as terminate threshold, and the preprocessed data was stored in the server in the form of a file. The mobile terminal equipment utilizes the Android 5.0 operating system. A native development kit was used to interact with the preprocessed images or the original image.

To measure the performance of the algorithm and the scope of the algorithm, the Jaccard coefficient was used to calculate the overlap with the ground truth [28], which can range from 0 to 1 with 0 indicating no overlap and 1 implies the perfect overlap. Defining similar indicator (SI) is as follows:

(5) $$\begin{array}{c} SI=\frac{S_{A}\cap S_{B}}{S_{A}\;\cup \;S_{B}}, \end{array}$$

where *S*_*A*_ is the segmentation result of the semiautomatic method and *S*_*B*_ is the manual segmentations by a radiologist due to the lack of a well-accepted ground truth.

In order to compare the performance of the method, the authors use the classical segmentation algorithms for comparative experiments. These algorithms include a classical level set method Chan-Vese (CV) [29], Snake [30], LuTA [15], and region growing. All the experiments are using the same origin data and using the same seed in LuTA and region-growing as used on the mobile side and initiate level set function around the seed by a circle with a diameter of 2 pixels and initiate the snake mask around the seed by 2 pixels.

### 4.1. Dataset and Segmentation Results

Experiments used data sets from the Henan Provincial People's Hospital, and the data set includes 129 cases of various types of glioma image. Besides, the data set has been hand marked and confirmed by two experienced radiologists. Through comparing the manual segmentation results and the semiautomatic segmentation results in this paper, the algorithm uses similar indicator defined by (5). After conducting the test, the SI can reach 75% over more than 80% of the samples. SI data is shown in Table 1.

**Figure 3:**
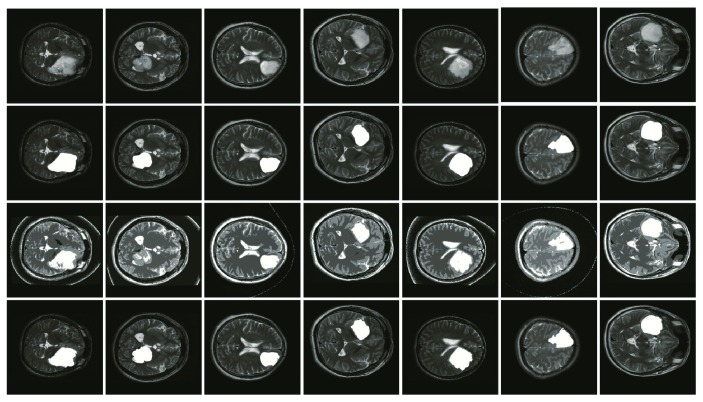
Glioma image segmentation results. Note: the first row is the original images, the second row is manually defined as ground truth labeled by a radiologist, the third row is segmentation results of HMICO, and the fourth one is obtained by the method of the present paper.

After examining the segmentation results, the method in this paper obtains good segmentation results with the images that have a relatively smooth morphology in the boundary. For these images, the segmentation results of our method are quite consistent with the manual segmentation, as shown in Figure 3.

For some of the cases that SI ≥ 50%, the performance of our method may be worse than those of the compared methods, but when examining these cases, our method is also found to have an acceptable result. For example, compared with that of the snake algorithm, the SI of our method is average less 13.42% with stand error of 7.69%, for the detailed data; see Table 2. For the cases that SI < 50%, the algorithm does not work, which will be discussed in chapter 4.5.

### 4.2. The Efficacy of Multiseeds and Hole Filling

**Figure 4:**
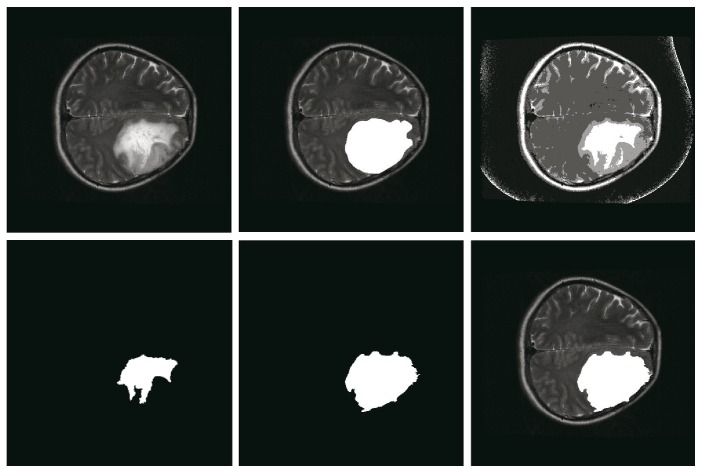
The effective of multiseed region growing. Note: the top row presents the original image, the ground truth labeled by a radiologist, and the result of HMICO from left to right. The bottom row shows the result of region growing with single seed, multiseeds, and the final result of our method, respectively. For this example, the SI of multiseed region growing can reach 87% while that of region growing with single seed is only 53%.

**Figure 5:**
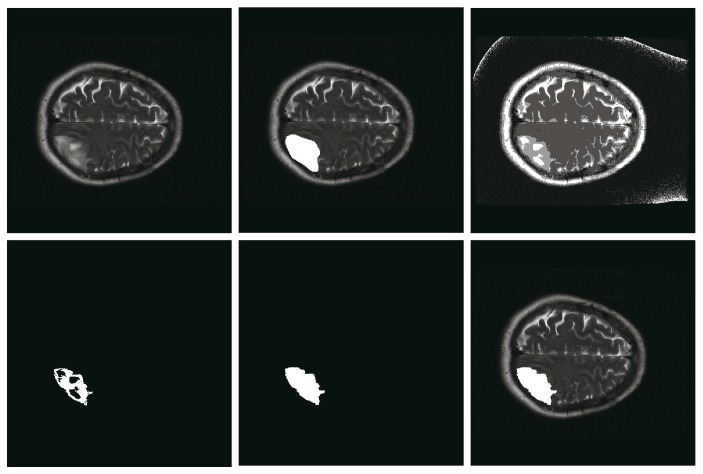
Schematic diagram of fill holes. Notes: the top row presents original image, the ground truth labeled by a radiologist, and the result of HMICO from left to right. The bottom row shows the result of region growing, the result of fill holes, and the final result of our method, respectively. For this example, the similarity of HMICO segmentation results (including holes) is 58%, while that after fill holes is 84%.

Due to the existence of strong tumor heterogeneity, the ROI may be divided into several subregions or contain little holes. For independent subregions, using multiseed region growing to merge them can improve accuracy. For example, the SI of multiseed region growing can achieve 87% while region growing with single seed is 53% as shown in Figure 4. For little holes within the ROI, hole filling can be used to solve it efficiently. For example, filling holes can improve the SI from 53% to 84% as shown in Figure 5. Compared with origin growing with single seed on all samples, the SI of our method with multiseeds and hole filling increases 2.57% on average.

### 4.3. Algorithm Reproducibility

Segmentation algorithm stability is one of the major challenges encountered during the implementation of radiomics, therefore guaranteeing the stability and reproducibility of segmentation results under the condition of guaranteeing that the acceptable accuracy is of more important significance.

The reproducibility of the segmentation algorithm mainly derives from two aspects. One is the iterations of the pretreatment algorithm; in order to improve iteration efficiency, this paper adopts the energy decrease rate threshold as the iteration-stopping condition; as uniform original parameters are adopted in the initialization, the same intermediate segmentation results can always be obtained for the same image. The other one derives from the selection of seed points; as the pretreated tumor region has same gray value, the same segmentation results can always be obtained through the algorithm in this paper, regardless of the position of seeds in the region of interest. Taking the above reasons into account, this algorithm has extremely high segmentation stability.

### 4.4. High Compression Performance for Mobile Devices

In order to realize real-time transmission, image segmentation, and image display of glioma on mobile equipment, the issues of real-time data transmission under low bandwidth wireless network, seed interactive selection on mobile equipment with small screen size, and completion of segmentation of the region of interest and real-time display on the screen under limited calculated performance of mobile equipment should be solved. All these issues have been excellently solved by the algorithm in this paper.

Moreover, since there are only four subregions in each processed image, the storage space can be extensively saved by coding each image. In practice, the data, preprocessed by HMICO on the server, is stored and transferred using unit 8 for a better implementation and reducing reconstructing time on the client. We tested our method on 1935 slices of 129 samples. Results show that the average size of the original images is 238.53 K, while the transferred result of HMICO is 22.18 K on average, which is about one-tenth of the original image.

Since the same region will get the same gray value after preprocessing, the algorithm will always obtain the same segmentation result when the seed is placed inside the ROI, demonstrating the robustness of our method. This has a considerable advantage, especially for mobile devices operated by a finger click on the small screen.

Since the data is preprocessed on the server, the time consumed by RegionGrow on the mobile is less than 0.01 s, which can be ignored. So, we only compare the computation time of MICO, LuTA, CV, and Snake on the server, as shown in Table 3. It can be concluded from Table. 3 that the average execution time of MICO is 1.86 s, the second best, and the standard deviation is 0.63, which demonstrates the stability of MICO.

### 4.5. Unsuitable Scenes

**Figure 6:**
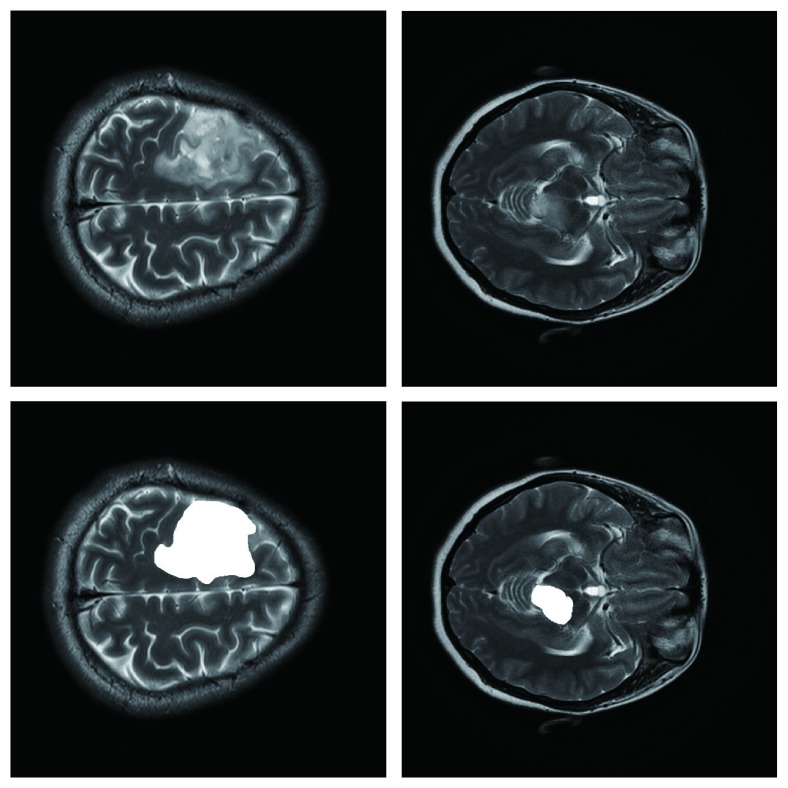
The scenes of algorithm do not work.

The algorithm does not work when ROI edges are blurred, or ROI presents a large number of the cross with other normal tissues, or ROI's gray level contains much overlapping with neighboring tissues.  Figure 6 shows two unsuitable scenes of the algorithm, the top row is the original image, and the bottom row is the corresponding segmentation result labeled by radiologist.

## 5. Conclusions

This paper adopts the modified hard edge multiplicative intrinsic component optimization algorithm for the pretreatment of glioma data, and the data are characteristic of small data volume and simple structure: thus, they can be excellently applied into a mobile equipment with low bandwidth, small screen, and limited calculated performance; the algorithm has excellent stability, which allows the doctors to conduct semiautomatic manual segmentation in their convenient time, and it is of excellent practical value.

Through analyzing the experiments, compared with manual segmentation, more than 30% of the sample can reach 90% similarity; over 60% of the samples can reach 85% similarity, and over 85% of the sample can reach 80% similarity, especially for the glioma images with a clear boundary. After being optimized for the glioma image, the algorithm obtains higher computational efficiency with better robustness.

The algorithm performance provided in this paper is poor for glioma images with a partially blurred edge and those having a large amount of gray overlapping with other normal tissues. Other targeted algorithms will be considered in the future work to solve such problems, and the automatic decision algorithm should be developed to further reduce the frequency of manual intervention.

## Figures and Tables

**Table 1:** The similar indicator of segmentations.

Algorithm	SI ≥ 75%		SI ≥ 85%		SI ≥ 90%	
	Number of samples	Proportion	Number of samples	Proportion	Number of samples	Proportion
Our method	109	84.50%	85	65.89%	42	32.56%
Snake	89	68.99%	75	58.14%	39	30.23%
RegionGrow	48	37.21%	34	26.36%	19	14.73%
LuTA	52	40.31%	38	29.46%	4	3.10%
CV	35	27.13%	22	17.05%	2	1.55%

**Table 2:** Comparison of SI on the cases that our method has worse SI.

Algorithm	Mean	STD	Max	Min
Snake	13.42%	7.69%	36.14%	0.01%
RegionGrow	8.02%	5.93%	24.67%	0.15%
LuTA	5.63%	4.29%	10.35%	0.09%
CV	8.95%	4.93%	19.44%	0.23%

**Table 3:** Execution time comparison.

Algorithm	Mean	Max	Min	STD
MICO	1.86	3.05	0.41	0.63
RegionGrow	0	0.01	0	0
LuTA	1.35	27.83	0.06	3.18
CV	3.81	6.21	0.79	1.28
Snake	2.82	3.89	1.33	0.57
